# Understanding the invisible workforce: lessons for general practice from a survey of receptionists

**DOI:** 10.1186/s12875-022-01842-4

**Published:** 2022-09-09

**Authors:** Ian Litchfield, Michael Burrows, Nicola Gale, Sheila Greenfield

**Affiliations:** 1 Institute of Applied Health Research, College of Medical and Dental Sciences, University of Birmingham, Birmingham, UK; 2 Department of Forensic Psychology, School for Health and Life Sciences, Coventry University, Birmingham, UK; 3 Health Services Management Centre, School of Social Policy, University of Birmingham, Birmingham, UK

**Keywords:** Occupational health, General practice, Triage, Patient access, Patient safety, Service delivery

## Abstract

**Introduction:**

The significance of the role of receptionists during the recent shift to remote triage has been widely recognised and they will have a significant role to play in UK general practice as it continues to cope with a huge increase in demand exacerbated by the COVID-19 pandemic. To maximise their contribution, it is important the social and occupational characteristics of the modern receptionist are understood, alongside their attitudes towards the role and their perceptions of the support and training they receive .

**Methods:**

We used convenience and cross-sectional sampling to survey the demographic characteristics of receptionists and various aspects of their role and responsibilities. This included the training received, specific tasks performed, job satisfaction, the importance of the role, and their interaction with clinical and non-clinical colleagues. We also captured data on the characteristics of their practice including the number of GPs and location.

**Results:**

A total of 70 participants completed the survey (16 postal and 54 online responses) of whom the majority were white (97.2%), female (98.6%), and aged 40 and over (56.7%). The majority of the training focussed on customer service (72.9%), telephone (64.3%), and medical administration skills (58.6%). Just over a quarter had received training in basic triage (25.7%). A standard multiple regression model revealed that the strongest predictor of satisfaction was support from practice GPs (β = .65, *p* <.001) there were also significant positive correlations between satisfaction and appreciation from GPs*, r(68) = .609, p < .001.*

**Conclusion:**

This study has provided a much-needed update on the demographics, duties, and job satisfaction of GP receptionists. The need for diversification of the workforce to reflect the range of primary care patients warrants consideration in light of continuing variation in access along lines of gender andethnicity. Training continues to focus on administrative duties not on the clinically relevant aspects of their role such as triage.

## Introduction

For decades the receptionist has been a constant at the forefront of general practice, and their role continues to combine the same key elements of routine administrative duties and facilitation of access to clinical care as it did at the inception of the National Health Service (NHS) [1–8]. Around them however, the primary care landscape has changed as the patients they deal with have become more ethnically diverse, tend to live longer and possess increasingly complex health needs [9]. General practice has also evolved, from its origins in numerous single-handed surgeries serving localised populations, toward fewer, larger, multi-disciplinary health care centres serving growing numbers of registered patients within an increasingly sophisticated health service [10]. These shifts have seen general practice become integral to the pursuit of a more equitable, integrated, and responsive NHS amidst expecations of an increasing reliance on tech-enabled health care [11–14].

As a result of COVID-19, what had initially begun in general practice as a measured process of tech-enabled service redesign suddenly became mandatory, rapid, and wholesale, particularly the dependence on tele-consultations and remote triaging [4, 15]. These fundamental changes in delivery have all been implemented without the recommended periods of consultation, and evaluation [4, 5, 16–20]. Despite being considered temporary at the beginning of the pandemic these processes are now expected to remain largely in place and cope with the unprecedented post-pandemic pressure placed on the service with receptionists at the heart of them [21, 22].

Despite the prominence and growing responsibilities of receptionists as a result of these new tech-enabled systems, they remain one of the least understood members of the general practice team [23] with much of our understanding based on research from previous decades [24–26]. Little is known of the characteristics of the current workforce [27, 28], or whether training or support is relevant or accessible. Without understanding receptionists’ needs, policymakers and practitioners run the risk of losing valuable patient-facing staff and misunderstanding the impact of recent re-design on the delivery of primary care [23, 28]. This article describes the results of a recent survey of general practitioner’s (GP) receptionists in England, providing a timely and apposite update on their socio-demographic characteristics, job satisfaction, relationships with colleagues, and attitudes towards the role and concludes with an exploration of the implications for post-pandemic general practice.

## Method

### Study design

A cross-sectional survey of reception staff across England and Wales was issued online and in hard copy to capture the demographic characteristics of receptionists and various aspects of their role and responsibilities including the training received, specific tasks performed, job satisfaction, their perceptions of the importance of the role, and the nature of their interaction with clinical and non-clinical colleagues. This survey was issued alongside a standardized work design questionnaire [29] the results of which have previously been published [30]. We also captured data on the characteristics of the practice where they worked including the patient list size and location [31]. Required responses were in the form of nominal (yes/no) answers, Likert scales, and checkboxes.

### Setting and sample

Sample size was calculated using a 95% confidence interval and a margin of error of 0.5. Based on existing population data [32] a sample size of 383 was necessary to accurately reflect the population of GP receptionists. All general practice receptionists in England were eligible to participate and they were sampled via convenience and cross-sectional sampling, with no exclusion criteria. The survey was designed to be distributed online hosted by Online Surveys UK (https://www.onlinesurveys.ac.uk/) and was supplemented by hard copies sent to a random sample of general practices via the post. The link to the online survey was disseminated via a number of relevant organisations which included Health Education England, every Clinical Commissioning Group (CCG), and a variety of newsletters and bulletins. Unfortunately, there is no overall contact list for receptionists in England with many receptionists only accessible via a generic practice email address. The survey was available for completion for 12 months from September 2016 until September 2017. Postal surveys were sent to 100 randomly selected practices from a list of all operating practices in the England [33]. In both online and postal surveys reminders were issued approximately 8 weeks after the original invitation was issued. In all cases, eventual participants were provided with an information sheet and signed an informed consent before completing the survey.

### Analysis

Data were exported from the OS system directly into SPPS (version no 24) or otherwise entered manually from the hard copies that were returned. The analysis included basic descriptive statistics and a multiple regression performed to understand the impact on job satisfaction of administrative duties, the key clinical role of overseeing repeat prescribing and the support received from practice GPs, as both administrative duties and repeat prescribing are key receptionist roles, and the importance of the support they receive from GPs is identified in the literature, [24, 34–37]. In order to explore the effects of the length of time in service on satisfaction, and the level of importance they attached to their role (and how appreciated they felt by GPs), a between subjects’ analysis of variance was performed on those who had been in the role for 0-5 years and those employed for greater than 6 years. This allowed us to explore the difference between short and long-term employment.

## Results

A total of 70 participants completed the survey (16 postal and 54 online responses) representing a broad geographical spread. Of those that responded the vast majority were white (97.2%), female (98.6%), and aged 40 and over (56.7%). They were typically heterosexual, (95.6%) and over half gave their religion or belief as Christian (51.5%). Two (2.9%) respondents reported having a disability. The majority of receptionists surveyed were educated to General Certificate of Secondary Education (GCSE) level (38.6%). Just over half (50.7%) had been in post for less than 5 years. hese characteristics are summarised in Table 1, some of which were reported in our previous paper that presented the results of a survey conducted in parallel that explored the job design of receptionists [30].

**Table 1:** Participant demographics*

Gender Identity *n*=70 (%)					
Female			Male		
*n*=69 (98.6)			*n*=1 (1.4)		
Age Range *n*=67 (%)					
18-28	30-39	40-49	50-59	60+	
*n*=14 (20.9)	*n*=15 (22.4)	*n*=11 (16.4)	*n*=20 (29.9)	*n*=7 (10.4)	
Marital Status *n*=69 (%)					
Single		Living with partner		Married/civil partnership	
*n*=26 (37.7)		*n*=9 (13)		*n*=34 (49.3)	
Disability *n*=68 (%)					
Yes			No		
*n*=2 (2.9)			*n*=66 (97.1)		
Sexual Orientation *n*=68 (%)					
Heterosexual	Gay/Lesbian		Bisexual	Other	
*n*=65 (95.6)	*n*=1 (1.5)		*n*=2 (2.9)	0	
Religious Belief *n*=68 (%)					
No Religion	Christian		Muslim	Other	
*n*=31 (45.6)	*n*=35 (51.5)		*n*=1 (1.5)	*n*=1 (1.5)	
Ethnic Background *n*=70 (%)					
White		Pakistani		Other	
*n*=68 (97.1)		*n*=1 (1.4)		*n*=1 (1.4; Italian)	
Highest Level of Education *n*=70 (%)					
No Qualifications	GCSE/CSE	Further Education	A-Levels	Batchelor’s Degree	Post-Graduate Qualification
*n*=3 (4.3)	*n*=27 (38.6)	*n*=19 (27.1)	*n*=11 (15.7)	*n*=8 (11.4)	*n*=2 (2.9)
Occupational Characteristics (*n*=69)					
Time in post *n*= 68 (%)					
0-5 Years	6-10 Years	11-15 Years	16-20 Years	21 Years +	0-5 Years
*n*=35 (50.7)	*n*=16 (23.2)	*n*=10 (14.5)	*n*=4 (5.8)	*n*=4 (5.8)	*n*=35 (50.7)

Our participants worked at practices of various sizes within diverse socio-economic environments with respondents working within each of the ten deciles described by the Index of Multiple Deprivation [38] broadly representative of the diversity of practices across England [39]. Similarly their practices were situated in locations reflecting a range of rural and urban locations with the majority working in the most urbanised areas reflective of the location of the majority of practices in England [39]. These characteristics are summarised in Table 2.

**Table 2:** Practice characteristics

Respondents Practice Size^a^ *n* = 69 (%)									
Small			Medium				Large		
*n*=4 (5.8)			*n*=38 (55.1)				*n*=27 (39.1)		
Number of GPs per practice									
1-5			1-5				1-5		
*n*=50 (71.4)			*n*=50 (71.4)				*n*=50 (71.4)		
Number of Reception staff									
0-10			0-10		0-10			0-10	
*n*=54 (77.1)			*n*=54 (77.1)		*n*=54 (77.1)			*n*=54 (77.1)	
IMD Score by decile^b^ *n*=34 (%)									
1	2	3	4	5	6	7	8	9	10
2 (5.8)	2 (5.8)	3 (8.8)	4 (11.8)	5 (14.7)	5 (14.7)	3 (8.8)	3 (8.8)	3 (8.8)	4 (11.8)
Rurality *n*=34 (%)									
Mainly rural	Largely rural		Urban with significant rural		Urban with city and town		Urban with minor conurbation	Urban with major conurbation	
2 (5.9)	6 (17.6)		6 (17.6)		5 (14.7)		2 (5.9)	13 (38.2)	
Response by region n =66 (%)									
Midlands	South East		South West		East		North West	North East	
*n*=32 (48.5)	*n*=11 (16.6)		*n*=6 (9.1)		*n*=5 (7.6)		*n*=9 (13.6)	*n*=3 (4.5)	

^a^As defined by Kelly et al [31]

^b^Indices of Multiple Deprivation [38]

### Receptionists’ duties

Participants were given a list of duties generally undertaken by the receptionist derived from existing research and asked to indicate which they considered to be their main duties, by ticking all that applied. Administrative tasks, arranging appointments, and dealing with difficult patients were amongst the most commonly reported, and the majority of our cohort also played a role in repeat prescribing and the reporting of test results (see Table 3). Additional tasks included, liaising with hospitals, pharmacies, and other external agencies, blood pressure checks, and chaperoning. Respondents were also asked to describe the roles they undertook that involved the need for medical knowledge or information. Just over half of the sample (57.4%) reported they would define some of their duties in this way (see Table 3).

**Table 3:** Main Duties undertaken by receptionist and self-reported clinically oriented duties

GP Receptionist's Tasks						
Number of respondents *n*=67 (%)^a^						
Administrative duties	Arranging appointments	Talking to patients (in any capacity)	Dealing with difficult patients	Repeat prescribing	Reporting test results	Other roles
67 (95.7)	67 (95.7)	66 (94.3)	63 (90.0)	43 (61.4)	42 (60.0)	31 (44.3)
Self- Reported Clinically Orientated Duties						
Number of respondents *n*=39 (%)^a^						
Triaging patients when booking appointments	Adding new medication (subject to GP approval), amending prescriptions	Reporting test results, changes in medication or diagnosis	Answering general medical queries, discussing medication with patients	Dealing with discharge paperwork	Chaperoning patients	Testing blood and urine
14 (35.9)	9 (23.1)	6 (15.4)	5 (12.8)	2 (5.1)	2 (5.1)	1 (2.6)

^a^Respondents were asked to tick all that apply so these may not add up to 100%

### Training

All but one respondent said they had received some training for their role, (98.6%) of these 56.5% (*n*=39) reported training both in-practice and by external agencies, 30.4% (*n*=21) reported only in-practice training and 13% (*n*=9) only external training (Table 4). The majority of the training focussed on customer service 72.9% (*n*= 51), telephone 64.3% (*n*=45), and medical administration skills 58.6% (*n*=41). Less than half had received training in medical terminology 42.9% (*n*=30) or basic triage 25.7% (*n*=18,). The most common barriers to training were lack of time 37.1%, (*n*=26) and funding 20%, (*n*=14) with other factors including a lack of support from practice managers 7.1% (*n*=5) or GP partners 5.7% (*n*=4). Just over a third, 38.3% (*n*=26) were either unsatisfied or very unsatisfied with the training they had received.

**Table 4:** GP Receptionists’ training

Have you received training *n* = 70 (%)				
Yes		No		
69 (98.6)		1 (0.4)		
If yes, was training internal/external *n*=69 (%)^a^				
Internal		External		
60 (85.7)		42 (60)		
Training Content N (%)^a^				
Customer Service	Telephone Skills	Medical Administration Skills	Handling Difficult Patients	Dealing with Complaints
51 (72.9)	45 (64.3)	41 (58.6)	41 (58.6)	38 (54.3)
Communication Skills	Medical Terminology	Assertiveness	Basic Triage	Other
38 (54.3)	30 (42.9)	24 (34.3)	18 (25.7)	12 (17.1)
Barriers to accessing training *n*=68 (%)^a^				
Lack of time	Lack of funding	Lack of relevant training	Lack of support from practice managers	Lack of relevant training
26 (37.1)	14 (20)	10 (14.3)	5 (7.1)	10 (14.3)
Satisfaction with training n (%)^a^				
Highly Satisfied	Satisfied	Neither satisfied nor Unsatisfied	Unsatisfied	Highly Unsatisfied
14 (20)	14 (20)	14 (20)	22 (31.4)	4 (5.7)

^a^Participants were asked to tick all that apply so percentages may not add up to 100

### Importance, satisfaction and appreciation

Receptionists were asked to rate how important they perceived their role on a Likert scale between 1 (highly important) and 5 (highly unimportant). The vast majority, 95.7 % (*n*=66) classed the role as very important or important, and just 2.8% (*n*=2) felt their role was unimportant or very un-important. Despite this nearly half of the sample were unsatisfied or highly unsatisfied (*n*=31, 44.3%) with their role. Respondents were also asked to provide a rating of satisfaction with elements of their job, selected based on the most important aspects of the role suggested by existing literature (Table 5). Overall respondents generally were highly satisfied or satisfied with administrative duties, triaging, support from practice managers and GPs, repeat prescribing and dealing with difficult patients. A total of 42.9% (*n*=30) felt appreciated or highly appreciated and 32.8% (*n*=23) felt unappreciated or highly unappreciated by their practice.

**Table 5:** Ratings of overall satisfaction, importance, sense of appreciation with the role and with different aspects of the receptionist’s role

	Highly Satisfied	Satisfied	Neither Satisfied nor Unsatisfied	Unsatisfied	Highly Unsatisfied
Overall Satisfaction with role *n*= 68 (%)	9 (12.9)	11 (15.7)	17 (24.3)	23 (32.9)	8 (11.4)
Administrative Duties *n*= 70 (%)	29 (41.4)	14 (20.0)	7 (10.0)	9 (12.9)	11 (15.7)
Triaging for urgent appointments *n*= 67 (%)	14 (20.9)	17 (25.4)	17 (25.4)	10 (14.9)	9 (13.4)
Support from practice GPs *n*= 69 (%)	19 (27.5)	13 (18.8)	17 (24.6)	10 (14.5)	10 (14.5)
Support from Practice Managers *n*= 70 (%)	16 (22.9)	18 (25.7)	12 (17.1)	12 (17.1)	12 (17.1)
Repeat Prescribing *n* = 58 (%)	19 (32.8)	13 (22.4)	12 (20.7)	8 (13.8)	6 (10.3)
Difficult Patients *n*= 70 (%)	13 (18.6)	15 (21.4)	23 (32.9)	13 (18.6)	6 (8.6)
	Very Important	Important	Neither Important nor Unimportant	Unimportant	Very Unimportant
Importance of the role *n* = 69 (%)	59 (84.3)	7 (10.0)	1 (1.4)	1 (1.4)	1 (1.4)
	Highly Appreciated	Appreciated	Neither Appreciated nor Unappreciated	Unappreciated	Highly Unappreciated
Appreciation *n*= 70 (%)	8 (11.4)	22 (31.4)	17 (24.3)	15 (21.4)	8 (11.4)

### Exploring satisfaction

The standard multiple regression model (Table 6) revealed that the strongest predictor of satisfaction was support from practice GPs (β = .65, *p* <.001). Table 7 shows significant positive correlations between satisfaction and appreciation*, r(68) = .609, p < .001*, as well as between appreciation and support from practice GPs *r(69) = .694, p < .01* and practice managers *r(70) = .665, p < .01*. These correlations imply that overall satisfaction and satisfaction with support from practice GPs and managers are significant factors in receptionists’ feelings of appreciation (see Table 7). Results revealed that there was little difference in satisfaction (*M* = 3.13, *SE* = .22) *F* (1, 64) = .00, *p* =.98 or appreciation (M =2.97, SE = .21) (M =2.75, SE = .21), F (1, 64) = .552, *p* =.46 over time. Results did however show those in post for 6 years or less perceived their role as less important (M = 1.44, SE =.93) compared to those in their role for more than 6 years (M =1.03, SE =.18), F (1,64) = 6.04, *p* < .05 (data not shown). This appears to indicate that their understanding of the importance of the role increases over time.

**Table 6:** Multiple regression model

Coefficients^a^							
	Unstandardized Coefficients		Standardized Coefficients	t	Sig.	Collinearity Statistics	
	B	Std. Error	Beta			Tolerance	VIF
(Constant)	1.609	0.297		5.415	0		
Administrative duties	0.109	0.117	0.135	0.927	0.358	0.492	2.033
Support from the practice GPs	0.566	0.115	0.645	4.923	0	0.603	1.658
Overseeing repeat prescribing	-0.101	0.12	-0.112	-0.843	0.403	0.587	1.703

^a^Dependent Variable: Satisfaction

**Table 7:** Correlational analysis

Correlations					
		Satisfaction	Support from the practice GPs	Support from the practice managers	Appreciation
Satisfaction	Pearson Correlation		.671*	.563*	.609*
	Sig. (2-tailed)		0	0	0
	N		67	68	68
Support from the practice GPs	Pearson Correlation	.671*		.800*	.694*
	Sig. (2-tailed)	0		0	0
	N	67		69	69
Support from the practice managers	Pearson Correlation	.563*	.800*		.665*
	Sig. (2-tailed)	0	0		0
	N	68	69		70
Appreciation	Pearson Correlation	.609*	.694*	.665*	
	Sig. (2-tailed)	0	0	0	
	N	68	69	70	

*Correlation is significant at the 0.01 level (2-tailed)

## Discussion

### General findings

Our survey found that modern day receptionists have retained the range of clinical and administrative responsibilities first adopted when general practice began [3, 7, 8]. Our participants were typically middle-aged and overwhelmingly white, heterosexual and female, though the response rate was lower than intended and we cannot assume our respondents are representative of the broader population of receptionists. Job satisfaction tended to be low and adversely impacted by a perceived lack of support from senior colleagues, with many reporting a lack of recognition of the value of their work. The training received tended to focus on administration, and communication or customer service skills, more so than the clinically relevant roles they performed.

### Strengths and limitations

The findings of this study have provided valuable and current insight into a group traditionally uninvolved in primary care research and service design. Although the respondents were drawn from practices from a range of rural and urban settings and socio-economic environments, the number of participants was below that anticipated (with only 34 respondents subsequently providing post codes). These low response rates could be due to the difficulties in obtaining individual receptionist addresses (whether email or postal) in the absence of a single national list, therefore much of the recruitment was conducted indirectly via senior GPs or practice managers. This means that we cannot be confident that our sample is truly representative, and our findings are transferable to every receptionist. In particular the small sample size may have also impacted on the number of respondents from ethnic minorities, exacerbated by the fact that Black, Asian and Minority Ethnic (BAME) groups are less likely to participate in research [40].

### Specific findings

#### Receptionist demographics

According to our survey and as observed in previous work, the vast majority of our respondents were female [24, 41, 42] seemingly in-line with the traditional supportive role of women in medicine [43] (it was only in 2014 that female GPs outnumbered their male counterparts [44]). The characterisation of the receptionist as a specifically female-gendered role is widespread [45] as was once the case with nursing [46]. It may be that the same reduction in stereotyping and gender bias that has seen numbers of male nurses increase [46–48] can be used to increase the number of male receptionists.

Discussing symptoms with receptionists has been identified as one of the major barriers to seeking care from GPs [49]. In this context the gender of the receptionist is an important consideration with evidence that some patients in primary care are more comfortable talking about their health with care providers of the same gender [50]. Receptionists are expected to ask specific questions around symptoms when booking appointments and understanding the impact of this apparent gender imbalance of reception staff on equitable access is an important consideration for researchers and policymakers; particularly when considering the comparative reluctance of men to seek medical help and its impact on prognosis [10, 51, 52].

Though the lack of BAME survey respondents may be attributed to the small sample it is important to note that the receptionist workforce would ideally reflect the ethnically diverse patient lists encountered at many English GP practices [53]. It is feasible that a lack of ethnic representation amongst reception staff could be a barrier to BAME patients accessing care [54, 55] due to a lack of culturally specific understanding [56] or unconscious bias towards ethnic minority groups [57–59].

#### Satisfaction

Though the study was conducted before the pandemic, and the mounting dissatisfaction with primary care that emerged in 2021, we found almost half our respondents were unsatisfied with their role, which was directly correlated to a perceived lack of support from senior colleagues. Since March 2020 the change in working practices meant many receptionists are now working in dislocated organisations. Previous work had described how reception staff felt ‘invisible’ to their colleagues [24, 27, 28] and that the complexity of their work and their contribution was misunderstood and under-appreciated [27, 28]. Satisfaction and appreciation underscore retention and staff turnover in healthcare [60] and given the current importance of the receptionist’s role during a period when general practice is weathering unprecedented pressure [21, 22] losing experienced reception staff would be of significant detriment to the service [61, 62].

#### Training

It has been understood for a number of years that receptionists traditionally undertake many clinically related duties without formal training specific to these roles [36, 63–66] and our results indicate that this remains the case. The sudden shift to teleconsultation-based care and mandatory remote triage that occurred as a result of COVID-19 meant receptionists were routinely remotely triaging patients, potentially making clinically relevant decisions every few minutes [1, 4, 16, 67]. This raises questions about how well-equipped receptionists are to undertake this remote triage and despite recommendations [68] there is a lack of evidence of the consistency of practice systems to support the role of receptionists performing this task [69]. Concerns remain over the effectiveness of strategies put in place for connecting with vulnerable patients and caregivers and those less technologically enabled or less able or confident to communicate via telephone or video call [20, 70–72]. As it stands there are widespread concerns that the exclusion of vulnerable groups has been accentuated by these rapid shifts in the modes of access to primary care delivered by staff without formal training [20, 69, 70, 73–75].

#### Future roles of receptionists

It appears that total triage and the increased reliance on remote access and digital connectivity is set to continue beyond COVID [76–78]. The growth in patients’ independent access to practice booking systems means the role of receptionists is likely to change. There is also an increasing use of online symptom checkers which can provide alternative diagnoses and suggest or facilitate a course of action including making an appointment with a GP [79], though early indications are that they tend to be favoured by younger or better educated patients [79].

The future role of receptionists is also likely to be impacted by the increase in the number of trained care coordinators [80] linking patients with appropriate resources and with responsibilities that include social prescribing [81]. Evidence of the clinical impact of these coordinators is mixed [82] and their introduction to existing systems is inconsistent [83]. However, patients report positive experiences [84] and the NHS is keen for the role to become more widely established in primary care [11, 81]. Despite such changes for those patients vulnerable to digital exclusion or otherwise from underserved communities mean it is likely that receptionists will continue to fulfil many of the core functions that have traditionally defined their role, and remaining as a key intermediary between patient and health service [75, 85].

## Conclusions

This study has provided a much-needed update on the demographics, duties and job satisfaction of GP receptionists. Although subsequently general practice delivery has changed as a result of COVID-19, the pandemic only served to highlight the potential fault lines resulting from relying on receptionists to perform their traditional range of duties without accounting for the evolving and increasingly complex patient body and NHS. It is important that the diversity of the receptionist workforce reflects those of the patients they serve to help minimise the differential access of care which can be observed along lines of gender and ethnicity. Considering the low levels of job satisfaction we found, the need to recognise, formalise, and support the role of receptionists in remote-triage and care navigation appears paramount. Failing to accomodate the attitudes, experiences, and perceptions of receptionists reduces the ability of general practitioners and policymakers to both optimise current care systems, and develop effective strategies for the future delivery of primary care.

## Data Availability

The datasets generated and/or analysed during the current study are not publicly available due to limitations of ethical approval involving the patient data and anonymity but are available from the corresponding author on reasonable request.

## Abbreviations

BAME: British and Minority Ethnic
CCG: Clinical Commissioning Group
GCSE: General Certificate of Secondary Education
GP: General Practitioner’s
IMD: Index of Multiple Deprivation
NHS: National Health Service
